# Collectanea Medica: Consisting of Anecdotes, Facts, Extracts, Illustrations, &c.

**Published:** 1819-08

**Authors:** 


### COLLECTANEA MEDICA:

CONSISTING OF

### ANECDOTES, FACTS, EXTRACTS, ILLUSTRATIONS, &c.

***Relating to the History or the Art of Medicine, and the Auxiliary Sciences.***

Floriferis ut apes in saltibus omnia libant,

Omnia nos itidem depascimur aurea dicta.

---

*Observations on the Excretion of the Milk.* (ŒUVRES DE BORDEU. *Récherches Anatomiques sur les Glandes,* § lxxiii.)

IT is now universally understood, that the excretory ducts of the breast terminate in considerable numbers in the nipple, where they are replicated on each other in such a manner, that, if they are extended or redressed by drawing out the nipple, they permit the milk to pass with increased facility.

It is also known that an infant, in the first instance, merely elongates the nipple by drawing it towards him, and the milk thus flows into his mouth; besides that, he may also, by sucking, draw the milk of the nurse who suckles him: but this is a peculiar species of excretion, on which we shall not here dwell. It has some analogy with the effect of a cupping-glass, and does not relate to my present object. Besides which, the mechanism of this is well explained in the *Memoires de l'Académie des Sciences.*

It should be remarked, that an infant, in sucking, besides extending the nipple, irritates or excites it; so that the breast itself enters into contraction, or into a sort of *erection.*

There is no nurse who has not experienced this tension, and a species of *titillation*, which is the consequence of it. They generally say, that they perceive the milk flow into the breast. The breast becomes round, firm, and distended; and there are some women who suffer drawing and tremulous sensations, which extend as far as the shoulders, the loins, and even into the arms. These sensations are painful in some women; but they ordinarily experience a titillation more or less voluptuous.

These irritations have so much influence on the excretion of the milk, that there are many mothers who cannot suckle any other than their own infant, because they are not excited by others.

The infant has difficulty sometimes in managing every sort of nipple; and nurses find children who do not excite them sufficiently, who do not induce a flow of milk, or who do not cause those *titillations,* or those *tremulous motions*, of which we have just spoken: but there is hardly any one who does not find some infant that suits her, to which she attaches herself the more, because it repays her by exciting sensations to which tenderness succeeds.

One might believe, when an infant sucks, and when he touches the breast, handling it in different ways, that he compresses it: the fact is, he elongates it a little, and excites it by friction.

There are some mothers who, when their infant touches them, are titillated to such a degree that a sort of constriction in their breasts takes place, which prevents the flow of milk. There are some that are less sensible, who say that the contact of the infant produces an impression or modification of sensation in their breasts, which they are unable to describe, and which does not differ from that species of *erection* of which we have already spoken.

It must be acknowledged, that there are some nurses in whom the milk escapes on compressing the breast: it makes a jet, but this jet is not of long duration; it only arises from the evacuation of the largest of the lacteal vessels situate near the nipple; and if the breast does not enter into contraction, the excretion of milk does not continue.

There are also some nurses who lose their milk in this way at certain hours, after taking food: their breasts have passed through all the states of which we have spoken, and the vessels have been so full, that the milk has escaped from a sort of disgorgement; but this only takes place to a certain point, and but little also flows from compression.

It is necessary to observe what passes here with attention; and, if we take care not to confound extension with compression of the breast, or with the changes which arrive in it by irritation, we shall be convinced that compression only causes the evacuation of part of the milk, which was contained in the largest ducts about the nipple, which are, as it were, little reservoirs, that they may be suddenly evacuated; but from which compression would never excite a continual flow of the fluid, without the causes which we have detailed.

We have seen women who have endeavoured to make their milk flow before the infant had sucked them, and put their breasts into action, but that has been impossible; but, as soon as the breasts had been thrown into contraction, by rubbing them and exciting the nipple, the milk has flowed of itself for a certain time, and could not be checked until the paroxysm subsided. This illustrates what we have stated above; and it is necessary to remark, that excitement of one breast is sometimes sufficient to put them both into action.

There are women who appear to have hardly any milk in their breasts, which are lax and empty; but, as soon as the infant excites them, they become distended, and the milk freely flows.

The history of the changes which take place in women in child-bed, and that of their maladies, demonstrates still better the species of contraction which we believe to be necessary in the breast, in order that the milk may flow from it. It is only necessary to pay attention to all these changes, to be convinced that the milk only appears in the breasts when they have been put into action; and it will be easy to perceive many phenomena which confirm it, that would occupy too much space in their detail.

Similar observations, which every person may readily confirm, and to which many additional peculiarities may be made, indicate the true cause of the excretion of the milk, which depends on a species of convulsive action, that, after having prepared the passages or canals terminating in the nipple, which has itself become tense, seizes the whole body of the breast, and disposes it to render its milk, when it is excited by the infant; which contributes, in its turn, to the excretion of that fluid by the suction it employs, as well as by the excitement of the organ which it produces.

---

COLLECTION OF RARE AND INTERESTING CASES.

[*Continued from vol.* xli. *p.* 311.]

" *Exudation of Blood through the Pores of the Skin.*—CATHERINE MERLIN, of Chaulney, in the 28th year of her age, received a blow from the foot of an ox on the epigastric region: she fell down insensible, and soon afterwards threw from the stomach a large quantity of blood. This hæmorrhage was renewed daily for a considerable length of time, accompanied with convulsive action of the stomach, notwithstanding the medical efforts that were made to arrest it. Her strength became much impaired, and at length the daily evacuation of blood ceased: it now occurred only after intervals of from eight to fifteen days. She then experienced a sense of fullness and heat about the stomach, vomited about a pound of blood, and then felt relieved. Hæmatemesis continued in this way during fifteen years, without any derangement in the menstrual evacuation taking place. An ignorant physician gave her strong astringent medicines to be taken internally; the vomiting then became partially suppressed, and an exudation of blood took place daily through the skin, from the extremities of the exhalent vessels. There is no part of the cutaneous surface that has not been the seat of this transudation. When the blood ceases to flow, the patient loses her appetite, feels oppressed and uneasy, and keeps in bed. This state of inconvenience continues to increase for some days; but an itching of some part of the skin announces that the hæmorrhage is about to occur, and when this takes place, all the accidents just mentioned disappear.

" When Catherine Merlin was forty-six years of age, she was seen by Dr. Boivin. She had ceased to menstruate two years; but this had not caused any change in the hæmorrhage from the skin. Twice a-day, at indeterminate periods, she feels a sense of heat and itching in some part of the skin, which is here a little tumefied; the blood escapes from the pores, and flows in large drops. On pressing the tumefied skin, which is painful, the escape of the blood is accelerated. After it has ceased, and the skin is washed, the surface does not differ in appearance from that of any other part of the body. This woman was in good health, and was not debilitated by the hæmorrhage: she habitually ate but little. From the colour of the blood, Dr. Boivin judged it to be arterial.—*Journal de Médecine*.

" I have had occasion," continues Dr. Fournier, " to observe a periodical hæmorrhage of the same kind, in a minister of state, forty-five years of age, who had undergone much anxiety and prolonged laborious study for several years. He was of the ordinary stature, had light hair, a reddish complexion, and a white and fine skin; he was of the sanguineous temperament, and of an impetuous character. After some long-continued midnight labours caused by an important subject, and at an instant when he experienced great mental uneasiness, he gave way to venereal indiscretions during part of the night. I found him the next day suffering great febrile agitation, with acute pains in the thighs, legs, groin, pubis, and especially in the penis. All these parts were inundated by blood: from the pores of the glans, in particular, it flowed in great abundance. This ceased under the use of some medical measures; but it has since continued to re-appear always after intervals of fifteen, or at farthest twenty, days, and has ceased, after two or three days, under the use of the same means. I have attended this patient during twenty months, and have regularly witnessed the recurrence of this accident: during the last three months, it has, however, been less considerable both in duration and in the quantity of blood thus evacuated. Acute pain has always accompanied the hæmorrhage, and the penis has constantly been the part most affected."

We refer our readers to the *Philosophical Transactions*, vol. v. for histories of three analogous cases, by M. Monginot, Dr. Mesaporitus, and Dr. W. Musgrave; which are not less curious and interesting than those above related by Dr. Fournier.

---

The following remarkable case of Paralysis, is taken from the "*Inductions Morales et Physiologiques*" of M. Keratry; a work which contains a multitude of highly-interesting facts, as well as judicious inductions, and which merits, in an especial manner, the study of the reflective physiologist.*

"There exists in the department d'Isle-et-Vilaine, an individual,† who, after having lost his sight for ten years, continued to be at the head of a financial administration. Persons who have negociated with him, have stated that he fulfilled the duties of his station in a very sagacious manner. A total deafness obliging him to resign his office, he confined himself to the transaction of the affairs of his family. He has been seen to communicate with the persons composing it, by means of characters placed on his hands. Although blind, he has had constructed, after his own designs, a mansion of an elegant style of architecture, the execution of which he superintended. A cruel reduction of his faculties did not prevent him modelling in wax a garden on a very convenient and agreeable plan; and he often rectified the proceedings of the person who executed it, solely by the accuracy of his sense of touch. After this, he suffered complete paralysis of the arms, lower extremities, and the external surface of the body. Stricken in his last moral relations, he would have ceased to exist to external objects, if it had not, on rubbing his cheek, been ascertained that this part still possessed sensibility. Then, in conformity with his desire, (for he had not lost his voice,) his attendants communicated with him by tracing characters on this part of his face, where the sense of touch still remained. Initial letters were sufficient, when questions relative to the common material wants of life were concerned. Abridgments were also formed for other objects of communication. This remarkable being, then, in his unexampled misery, still possesses some

---

* Mr. Souter, of St. Paul's Church-yard, has at present a few copies of this work.

† M. Judicelli, ancien directeur des droits réunis à Rennes.

tender sensations from external relations. The hand of his wife, that of his daughter, or of a friend, makes his heart throb by sure though singular indications. Reduced to the actions of the pulmonary organs and the assimilating and digestive tubes, he claims, on the verge of the grave, and with a sort of success, the distinctive character of man,—intellectual intelligence. As the absolute immobility of his posture seems to involve in impenetrable shadows the other acts of his existence, we are led to view in him, when he speaks, a living wreck of brain alone."

---

### *Observations and Reflections on Vital Action.**

"All the phenomena of life may, in ultimate analysis, be reduced to two simple facts: a *sensation* of the presence of foreign bodies, when brought into contact with the organized tissue; and a *re-action*, by which the tissue, and in some cases the foreign body also, undergoes a consequent alteration.

"The term sensation, it should be premised, is employed by physiologists in a peculiar sense. To explain its use, it must be understood, that all bodies do not act indiscriminately upon every living tissue; each particular organization being so constituted, as to stand in relation with its own peculiar series of agents, by which alone it can be congruously affected. These agents are called the *stimuli* of the organization.

"The action, which results from the presence of a stimulus, being neither chemical nor mechanical, the tissues are considered as feeling or distinguishing between the different substances applied to them. Some affection of the living tissue must result from external impact, to determine its action or quiescence: this affection, inasmuch as it depends not upon the inorganic properties of the tissue, but arises out of its vitality, requires a distinct appellation; and from analogy it has been termed a sensation.

"In all tissues, unconnected with a nervous apparatus, these phenomena take place independently of consciousness; and, as they occur in beings altogether divested of nerves, they cannot be considered as connected with perception. Sensibility, therefore, in this restricted use of the word, represents merely the unknown modification of the tissue, which causes it to re-act, or not to re-act, according as the substance applied is, or is not, one of its appropriate stimuli. In this there seems to be little more than an habitude of the tissue with respect to its stimuli, analogous to that which in chemistry is termed *elective attraction*.

"This analogical use of the word 'sensation,' is perhaps scarcely justifiable, on account of its obvious tendency to mislead the imagination. Its close etymological connexion with the organs of sense, which must at every turn suggest the notion of consciousness, renders it a very unsafe term, when used to express a force independent of perception; a force which exists in vegetables, in amputated portions of animals, in eggs, and in seeds.

"Relative sensibility is that modification of living power which is

---

* From Sir T. C. Morgan's *Sketches of the Philosophy of Life.*

attended by consciousness, and appears to have its exclusive seat in a nervous apparatus: it ought, perhaps, on this account, rather to be considered as a function than as a primitive force.* But, the distinctions between tissue and organ, vital property and function, are too arbitrary to admit of an universal and rigid application; and utility alone must decide upon their individual appropriations.

"To express the re-action which takes place in the living tissues, upon the application of their appropriate stimuli, physiologists have adopted the term *contraction:* in this, as in the preceding case, deriving their notions from the more obvious movements which occur in animal tissues of the highest order.

"The precise nature of the simplest mode of organized re-action, which constitutes *nutritive, automatic,* or *organic* function, is perfectly unknown. It takes place in portions of the living solids, too minute to be submitted to the test of ocular observation. A particle of blood may be traced in its course through a vessel; it may be seen entering a gland, from which it escapes totally altered in its constitution, under the form of a given secretion; but the nature of the process which it has undergone escapes the scrutiny of the senses, and is wrapped in the most impenetrable obscurity.† In like manner, the food may be traced through every stage of the digestive process, and its progressive alterations noted; but, the peculiar action of the living alimentary tube, is not rendered in the least manifest by the observation. It is from analogy alone with the sensible contractions which take place in the fibres of muscles, that these various imperceptible movements are designated by the term contraction.

"Bichât, the original author of this mode of considering vitality, recognizes three varieties of organic re-action. The first, which he calls *organic insensible contraction*, but which is better designated by the terms automatic or nutritive contraction,‡ includes all the minute vascular actions of assimilation and absorption, which, being independent of volition, are conducted by causes inherent in the organs themselves.

"His second variety he terms *organic sensible contraction*, which embraces those manifest muscular movements that take place in organs exempt from the influence of volition, such as the heart and muscular coat of the intestines.

"The last variety he has named *animal contraction,* including by the term all those muscular movements which are directed by the will. This arrangement, however, is evidently inadmissible; inasmuch as it is founded upon a distinction extrinsic to the subject. Both the last modes of contraction are perfectly alike, and are included by Haller under the appellation of *irritability*. The circumstance of a muscle being connected, or not connected, with the nervous apparatus, and thereby being subservient or independent of volition, is utterly foreign

---

* "Magendie *Précis de Physiologie.*

† "The passage of an electric shock, as has already been observed, is insufficient to explain the differences of secretion.

‡ "The use of the term '*organic.*' as opposed to *animal* action, is vicious; inasmuch as all living actions are organic. It involves likewise the odious cacophony of organic organs.

to its structure as a muscle; and, if the nervous communication between the brain and an organ of animal contractility be cut off, its muscular properties still remain entire and unaltered.

"There are, it is true, some slight differences between the mechanical arrangement of the fibres of those muscles by which organic movements are executed, and those which obey the influence of the will, which it is necessary for the physiologist to bear in mind; and for this purpose, Bichât's discrimination is not without its convenience: but it must always be remembered, that the two forces are so much alike in all the different cases, that there are but slight philosophical grounds for establishing a separation of their phenomena into separate and contrasted orders.

"The several distinctions here attempted, both between the modes of contraction and of sensation, should indeed be regarded as in a great measure artificial; their value depending rather upon their utility in the processes of generalization, than upon their close approximation towards philosophical accuracy.

"Every distinct tissue possesses its own specific vitality; and the differences of sensibility which give birth to a flower or a muscle, must far exceed those which make the distinction between automatic and animal function. That the varieties of sensibility established by Bichât consist rather in degree than in kind, seems evident likewise from the sudden development of the perceptive sensibility during the inflammation of organs, divested of this faculty in their healthy condition. In like manner, the transition from the insensible contractions of nutritive function to the visible action of voluntary muscles takes place by such fine shades, as have involved physiologists in endless disputes concerning the muscularity or non-muscularity of particular organs.

"In tracing the phenomena of nature from the highest and most complicated movements of volition, down to the simple gravitation of matter towards a common centre, it is difficult to perceive the points at which the influence of one modification of force ceases, and another commences.

"There is every reason, short of positive proof, for supposing all the phenomena of life to depend upon one common principle, variously developed in the different living tissues. Between the lowest exertions of this principle and the cases of mere chemical affinity, the differences are scarcely appreciable; and the resemblance between chemical affinity and the attractive force of masses, is still more striking.

"So strictly similar is the mode of operation of all these different forces, that language affords but one common term for the sympathetic solicitations of love and friendship, and the mutual action of unorganized bodies. It is on this account that the mystic philosophers of antiquity attributed all things to the divine Eros,* and that Lucretius commences his system of nature by an invocation to Venus.

---

"It must however be repeated, that the hypothesis arising out of this view (beautifully grand as by its extreme simplicity it is) belongs, in the present state of our knowledge, rather to the province of poetry than of physiology.

"The sensibility and contractility of organized tissues, not being inherent in their ultimate particles, are developed by the functional actions which take place between the solids and fluids; and the suspension of these actions is speedily followed by death.*

"The influence of the circulation upon the sensibility of relation, is still more immediate. If the blood by any means be prevented from flowing in its accustomed quantity towards the head, the perceptive functions are instantly suspended; the animal faints; and, unless the cause be speedily removed, syncope is exchanged for death. If, on the other hand, the circulation continue uninterrupted, and the ligature be cast round the nerves of a limb, so as to cut off its communication with the cerebral centre, the other tissues will continue their functions, uninterrupted by the accident. An injury having been inflicted upon the nervous apparatus, the communication of sensations to the brain, and of volition to the muscles, will indeed be of necessity suspended; but the limb will continue to live, and its muscles to contract, in obedience to other stimuli, as if no such operation had been performed. These counter-experiments clearly demonstrate that the nervous system is not the fountain of life to the rest of the economy, but receives its animation, in common with all other tissues, from the action between its own vessels and the circulating fluids.

"The manner in which the blood is distributed to the different organs, is in strict agreement with this conclusion; the quantity which flows to any one of them being closely commensurate with the intensity of its functions. One-sixth of the whole circulating fluids has been calculated to pass through the brain, in the human subject; and the muscles, which are the parts most liberally supplied with nerves, have likewise the largest quantity of blood-vessels in their structure.

"Vitality being thus at once the offspring and cause of function, there is exhibited a circle of actions, which affords neither commencement nor termination, and in which the primary cause is hidden from contemplation. The renewal of the vital energy is, however, in some way connected with the function of respiration. The blood of the mammalia, in vivifying the solids, undergoes that unknown change, which, while it confers upon it its purple colour, renders it unfit for the repetition of its functions. A counteracting process takes place in the lungs, and the energizing property of the blood is restored. The chemico-vital action of the air in these organs, is therefore demonstrably the source of regenerated vitality."†

---

Λυσιμελὴς πάντων τε θεῶν πάντων τ' ἀνθρώπων
Δάμναται ἐν στήθεσσι νόον καὶ ἐπίφρονα βουλήν.

HESIOD *Theogonia.*

* This indeed follows from the very meaning of the term; life is essentially action.

† We have omitted some interspersed passages containing illustrations of the preceding propositions, from their being familiar to professional readers in general.—EDIT.

